# Evaluating primary care transformation: synthesis of findings from UK pilot project reviews

**DOI:** 10.3399/BJGPO.2022.0154

**Published:** 2023-05-17

**Authors:** Emilie McSwiggan, Lauren Ng, Eddie Donaghy, Huayi Huang, John Gillies, David AG Henderson, Andrew Thompson, Harry HX Wang, Stewart W Mercer

**Affiliations:** 1 Advanced Care Research Centre, University of Edinburgh, Edinburgh, UK; 2 Usher Institute, University of Edinburgh, Edinburgh, UK; 3 School of Public Health, Sun Yat-Sen University, Guangzhou, China

**Keywords:** primary health care, general practice, transformation, evaluation, new models of care, pilots

## Abstract

**Background:**

Pilot 'new models' of primary care have been funded across the UK since 2015, through various national transformation funds. Reflections and syntheses of evaluation findings provide an additional layer of insight into 'what works' in transforming primary care.

**Aim:**

To identify good practice in policy design, implementation, and evaluation for primary care transformation.

**Design & setting:**

A thematic analysis of existing pilot evaluations in England, Wales, and Scotland.

**Method:**

Ten studies presenting evaluations of three national pilot studies — the Vanguard programme in England, the Pacesetter programme in Wales, and the National Evaluation of New Models of Primary Care in Scotland — were thematically analysed, and findings synthesised in order to identify lessons learnt and good practice.

**Results:**

Common themes emerged across studies in all three countries at project and policy level, which can support or inhibit new models of care. At project level, these included the following: working with all stakeholders, including communities and front-line staff; providing the time, space, and support necessary for the project to succeed; agreeing on clear objectives from the outset; and support for data collection, evaluation, and shared learning. At policy level, more fundamental challenges related to the parameters for pilot projects, in particular, the typically short-term nature of funding, with an expectation of results within 2–3 years. Changing expectations about outcome measures or project guidance part-way through project implementation was also identified as a key challenge.

**Conclusion:**

Primary care transformation requires coproduction and a rich, contextual understanding of local needs and complexities. However, a mismatch between policy objectives (care redesign to better meet patient needs) and policy parameters (short timeframes) is often a significant challenge to success.

## How this fits in

By analysing lessons learnt from syntheses of evaluations of primary care transformation projects in the UK, this review further builds the understanding of factors that contribute to or inhibit the success of pilot projects at a local or regional level. It adds a level of insight into common factors at the policy level that support or impede success. Many primary care clinicians will be involved with transformation projects as this remains an area of policy action across the UK. This review provides useful information that may assist with project planning and delivery, and also with recognising potential opportunities and challenges in terms of the overall policy context.

## Introduction

Since 2015, against a backdrop of growing pressure on general practice — driven by greater overall use of primary care and increasing numbers of people with multiple comorbidities and complex care needs^1^ — national governments across the UK have set out multi-year plans for investment in primary care development.^2–5^ These investment plans are linked together by the concept of 'transformation', that is, providing additional funding and forms of non-financial support, on a time-limited basis, to facilitate the development of new working models within primary care, which better meet the needs of patients while reducing pressure on the health and social care system as a whole. This has resulted in the creation of many local and regional pilot projects across England (Vanguards), Scotland (National Evaluation of New Models of Primary Care in Scotland), and Wales (Pacesetters).

As a condition of funding, these pilot projects are usually required to be evaluated. Numerous local evaluations and several large-scale national evaluations have already taken place.^3,6,7^ Approaches to primary care across the UK are still evolving, with governments continuing to invest in plans for further service or system redesign.^2,8–10^ However, differences exist in the way in which policies are delivered across the four nations.^11^

This study considered the following three major UK primary care transformation programmes: Vanguards (England); Pacesetters (Wales); and National Evaluation of New Models of Primary Care in Scotland (Scotland). Launched by NHS England in 2015, the Vanguard programme aimed to provide patients with more personalised and coordinated care, by transforming working relationships within and between primary, acute, and emergency care.^12^ There were 50 Vanguard sites — discrete local or regional areas of health service provision — which were funded to trial new ways of working, based on their own analysis of local needs. These were supported by a £200 million transformation fund and a central National Support Programme to implement and evaluate their projects.^12^ Projects were centrally funded and evaluated for a maximum of 3 years.^12^ The Welsh Government allocated £4 million to pilot innovations in primary care (known initially as Pacesetters and Pathfinders, then just Pacesetters), from 2015–2018,^3^ with further funding tranches released in 2018, 2020, and 2022.^13^ Initial funding was allocated proportionately to all Welsh health boards by share of population.^3^ Some centralised support for implementation and evaluation was provided through Public Health Wales.^3^ The Scottish Government established a Primary Care Development Fund in 2015, providing £30 million of funding to trial new models of primary care.^6^ These 'tests of change' took place in every Scottish health board and were diverse in their design and focus in response to local needs or priorities.^6^ There was no centralised support for planning and implementing projects, but a nationwide evaluation took place after the conclusion of the pilot phase.^6^ Projects were funded for 2 years, from April 2016–March 2018.^6^

Across all three programmes there was a common expectation that if pilot projects were successful they would be 'mainstreamed' by the responsible health provider;^3^ meaning that they would be funded from that provider’s core budget once the period of additional project funding ended. While a similar primary care transformation programme was launched in Northern Ireland in 2018,^2^ progress so far appears to have been impeded by funding and governance challenges,^14^ and evidence of any project evaluations being published to date has not been found.

The aim of this study was to synthesise learning from the existing evaluations of these three national programmes, in order to identify the opportunities and challenges presented by various approaches to primary care transformation, and to identify areas of good practice and recommendations for future policy and practice.

## Method

The following three distinct national programmes of primary care transformation have taken place in the UK since 2015: Vanguards in England; Pacesetters in Wales; and National Evaluation of New Models of Primary Care in Scotland in Scotland.

In each of these programmes, various pilot projects were carried out in different primary care sites across the country. Pilot projects were typically envisaged as having the following three stages: planning; implementation; and evaluation. Thus, many of the individual pilot projects were evaluated at the end of their funding period, either by the project team or by independent evaluators. These evaluations, carried out at the level of the individual project, are referred to as 'first-level' evaluations.

A 'second level' of project evaluations was further identified. These either undertook a combined evaluation of multiple pilot projects^6^ or synthesised findings from multiple existing evaluations.^3,7,12,13,15,16^ These differ from the first-level evaluations because they draw together a range of pilot projects and identify common themes, which could inform lessons learnt or recommendations for policy and future good practice; and, uniquely, they allow the effectiveness of the project-level evaluations to be compared and scrutinised.

This review compared and thematically analysed the 'second-level' evaluations of primary care transformation programmes in the UK. The study began by including the nationally commissioned evaluations of the three programmes.^3,6,7,12,13,15–17^ This was supplemented with additional searches for independent academic or grey literature through scoping searches on Google, PubMed, and key government and public sector websites for the UK nations, as well as hand-searching the reference lists of included studies for further relevant studies. The review included any studies that conducted multiple evaluations of primary care transformation projects in one of the three UK programmes; or which synthesised multiple evaluations; or which reflected on or conducted additional analysis of ≥1 existing project evaluations.

The studies were analysed using a thematic approach. For each study, lessons learnt and policy recommendations (regardless of whether these were included in a formal 'conclusions' section or part of the main body of the text) were extracted and these were grouped together by theme for analysis. Data extraction and identification of themes was done by three authors. One further author independently read the identified studies and considered whether the themes identified by one of the three authors were accurate and whether any important issues had been missed. The four authors then discussed and agreed on the final themes included.

## Results

Ten studies were identified that met the inclusion criteria. Seven of these related to Vanguard initiatives in England; two to Pacesetter initiatives in Wales; and one to National Evaluation of New Models of Primary Care in Scotland in Scotland. A brief overview of relevant study characteristics is included in Table 1. From these studies, five themes were identified, which were further divided into 11 subthemes, set out in Table 2. These are described in more depth below.

**Table 1.** Summary of characteristics of included studies

First author	Date	Location	Focus
Checkland^15^	2021	England	**Qualitative reflection** on performance metrics and evaluation from six Vanguard sites
Checkland^16^	2019	England	**Evaluation** of the national support programme for Vanguard pilots, including the approach to local and national evaluation
Evans^13^	2022	Wales	**Summary** of lessons learnt from evaluations of 2020–2022 Pacesetter projects
Fowler Davis^19^	2020	England	**Reflection** on a local evaluation of one Vanguard site
Laverty^17^	2019	England	**Reflection** from 12 STP leaders on the Vanguard projects within their areas
McCarthy^22^	2019	England	**Comparison** of one rapid and one longitudinal evaluation of a Vanguard project
Mercer^6^	2019	Scotland	**Thematic summary and recommendations** based on evaluation of 204 new models of primary care
Miller^3^	2018	Wales	**Critical appraisal** of Pacesetter programme (24 initiatives; 156 study participants)
Starling^18^	2017	England	**Qualitative** overview of learning from 45 leaders within eight Vanguard sites
Wilson^7,12^	2019 and2021	England	**Synthesis** of 115 local Vanguard evaluations and **qualitative** exploration of evaluation leads’ experiences of evaluation

STP = sustainability and transformation plan.

**Table 2.** Themes identified from the included studies

Theme	Subtheme
***Factors that contribute to …***	***Include …***
A. Making the right kind of changes	1. Design and planning
	2. Values and priorities
	3. Involvement of patients and communities
B. Managing change effectively	4. Organisational context, protected time, and capacity building
C. An enabling environment for change	5. Decision makers and stakeholders
	6. Key parameters: funding and timing
D. Effective evaluation	7. Availability of data
	8. Quality of evaluation
	9. Standardised approaches
E. Applying what has been learnt	10. Opportunities for shared learning
	11. Receptive policy environment

### Making the right kind of changes

#### Design and planning

'*Taking time to understand and adapt to the local context is essential for new care models*.^18^'

Conceptualising and designing projects carefully provides a strong foundation for their future success. Studies identified factors that contributed to an effective design stage, and factors that inhibited it, at project and at policy level.

Enabling factors included involving stakeholders who could provide *'significant insight'* ^18^ into the needs of patients and populations, from the very start of the design phase. This included patients and communities themselves, as well as primary care providers^18^ and front-line staff.^6^

Additionally, a clear and well-documented project plan, established at the outset, can contribute positively to project implementation and sustainability.^3,6^ A significant dimension of this was establishing the project’s aims and objectives at the start,^3,12^ so that success can be evaluated in due course, and so that early conversations can be had in which assumptions are tested about how the project will achieve its desired results.^18^ Conversely, lack of consistent project management support was identified as a barrier for some pilot projects.^13^

In this context, studies identified the importance of planning for evaluation (and involving evaluators in planning) from the outset of new projects.^12,13^ This would allow outcome measures to be agreed that reflect the project’s objectives,^19^ and arrangements to be made to collect appropriate data.^6^ Several studies identified this as an area for improvement in practice.

In England, Vanguard projects were supported to develop logic models as a condition of their first year of funding. The use of logic models was a critical first step in designing, planning, introducing, and assessing local change on the ground,^7,12,18,19^ and could have been a key feature of effective design and planning if used consistently. In practice, however, evaluators of individual projects rarely used these logic models or even referred back to the impacts originally proposed in them.^12,16^ This gap between initial design and objective-setting, and the measures that were eventually used to evaluate projects, was mirrored at policy level; for example, Checkland *et al* ^15^ reported that, while Vanguards could (and did) initially set their own objectives, these were superseded by the introduction of national metrics part-way through the project.

#### Values and priorities

*'Population health outcomes remain elusive, in spite of the original commitment*.*'* ^19^

Several studies found a gap between the stated aims of the transformation funding and the types of project that were actually funded. In Scotland, despite an original expectation that *'every proposal will make clear how it intends to address health inequalities'*, this was only reflected in 10% of the projects that were actually funded.^6^ Likewise, while the funding call encouraged proposals to address *'equity of access to services'*, evaluation found that projects did not adequately understand or address the differing needs of rural communities.^6^

At project level, this seems to indicate a lack of readiness to address these more complex and structural issues as part of the design of transformation projects. While this might be owing in part to the limited time and resources available for those projects, evaluation suggests that there are also knowledge gaps at the level of service providers (in terms of health inequalities in Scotland, this could be filled by *'existing evidence, and learning from "GPs at the Deep End"'*, as well as greater understanding of how to apply *'rural proofing ... as a systematic approach'*,^6^ which, if addressed, could enable more inclusive design even within relatively short-term projects.

At policy level, the gap between the originally stated values and the projects that were actually funded suggested either limited policy commitment to those values, or the absence of a mechanism that enables funders to select projects that reflect the full range of policy priorities.

#### Involvement of patients and communities

*'Patient, carer, and community involvement is essential*.*'* ^6^

Studies identified the involvement of patients, families, and communities in all phases of the project, including design, delivery, and evaluation, as key to project effectiveness and as a significant area for improvement in practice.^3,6,12,18^ At a policy level, this could be addressed by making patient and community involvement a condition of funding,^3,6^ as it is for most UK research funding,^20^ and by developing national and local infrastructure for patient involvement.^3^

One of the major limitations on patient involvement in practice appeared to be time,^12^ which is something that might perhaps be mitigated in part by focusing initially on a clear and fairly narrowly defined population^18^ or by extending the project timeframe.^6^ A study of patient and public involvement in primary care research found that this is still relatively limited in scope and quality, and recommended ongoing work to improve researchers’ (as well as patients’) skills.^21^ If this is true of research, it is reasonable to consider that health and care professionals may also benefit from further training to develop the skills necessary for effective patient and public involvement in service transformation.

### Managing change effectively

While the themes discussed above relate to planning effectively for the project itself, this theme related to planning for success in terms of the context in which the project would take place.

#### Organisational context, protected time, and capacity building

*'Sites described taking a pragmatic approach … identifying clinical individuals and teams that already had ideas for and commitment to change.'* ^18^

Several studies identified the value of using existing foundations within organisations:

*'Tests of change that built on previous work and where pre-existing relationships were functional, were implemented more effectively than those that were entirely new.'* ^6^

Starling^18^ described this as *'go*[ing] *where the energy is',* which refers to working with teams that already want to make changes, as a way of gaining momentum, building local leadership, and avoiding burnout of teams as a result of top-down imposition of ideas.

Alongside this, building organisational capacity for change by providing time, training, and support for all those involved with the project was a recurring finding.^3,6,18^ Having sufficient protected time and the infrastructure support to discuss, plan, and deliver change on the ground is crucial to successful projects, and *'some sites expressed frustration with the compressed timetable set by the national programme'*,^18^ which was recognised as a limit on their ability to deliver transformation projects effectively.

In addition to providing protected time for planning and delivering the project, the importance of time and space for reflective learning was also indicated.^3^ Creating a dedicated team to support the project,^3,18^ building trusting relationships across boundaries,^16^ and putting in place inclusive project governance structures^3^ were other forms of organisational infrastructure that contributed towards the chances of a project’s success.

### An enabling environment for change

#### Decision makers and stakeholders

*'Role clarity, role support, governance, and clear communication channels are required as the primary care landscape becomes more complex*.*'* ^6^

At the policy level, decision makers have an important role to play in creating an enabling environment for change. The two main challenges that studies identified in this respect were clarity and consistency.

Clear roles, responsibilities, and channels of communication were important to a project’s success.^6^ Clear and consistent parameters were also essential; for example, studies identified particular challenges related to outcome metrics being changed after projects had started^15^ and national guidance for evaluations changing after evaluations had begun.^12^

Effective engagement with internal and external stakeholders is also a key factor in project success. Within projects, this can take the form of cross-organisational forums for decision making, provided that forum attendees are seen as credible by those they are representing.^18^ It can also mean involving people at different levels within organisations, and with different roles, in the decision-making process.^6,18^ Externally, engaging with professional bodies and networks may be necessary to ensure they understand and support the change.^3^

#### Key parameters: funding and timing

*'Whether the Vanguard initiatives were locally re-commissioned often depended not on the Vanguard success — or how well the evaluations captured Vanguard success — but rather on available finances*.*'* ^12^

The availability of funding and the timeframe within which projects are expected to demonstrate impact are two key parameters set at the policy level, which have a significant impact on the actual or apparent success of a project.

Most primary care transformation projects covered by these studies were funded by short-term (that is, 2–3-year) funding arrangements, usually with an expectation that projects would either be independently sustainable after that point^3^ or be prioritised against other initiatives within local budgets;^12^ or would perhaps have demonstrated sufficient benefit to make the case for ongoing national funding.

However, studies identified significant limitations in respect of these timeframes. They did not allow for messy realities, such as delays in procurement processes^12^ and iterative redesign of projects,^19^ which would have impacted on the timetable for projects to deliver outcomes. There was *'a very real tension between a narrative that emphasised long term and meaningful "bottom up" change and one which required the demonstration of results within a timetable, which satisfied the political needs associated with the programme'*.^16^ Overall, a 2–3-year timeframe may simply not be long enough for projects to demonstrate the kinds of outcome that they were designed for.^6,13^ McCarthy *et al* ^22^ compared two evaluation timeframes for a single project, finding that the short-term model substantially overestimated the costs and underestimated the savings compared with a longer-term evaluation of the same initiative.

### Effective evaluation

#### Availability of data

*'The lack of access and availability of useful primary care data was cited as a barrier to making evidence based and measurable changes in primary care*.*'* ^13^

At the evaluation stage, the ability of projects to demonstrate impact was hampered by a lack of complete, high-quality, and relevant data.^6,12,16^ As discussed above, this demonstrated the need for outcomes to be agreed, and approaches to evaluation to be considered, as early as the design stage of the project.^3,12,18^ Organisations need to be able to collect data that are fit for purpose and reflect population-level as well as service-level objectives.^19^

#### Quality of evaluation

*'Although a majority* [of evaluations] *state intentions to capture patient experience and conduct "economic" or "cost" related analysis, a combination of resource, data, time constraints mean that these components often lack depth, are often not fully realised or not conducted at all.'* ^12^

Although a commitment to evaluation was designed in to most transformation funding, the studies found that the quality of evaluation was not necessarily robust. Limitations on time meant that evaluations were limited in scope;^12^ local evaluation teams struggled with access to data and key informants,^12,19^ shaping appropriate evaluation questions, and methodology.^12^ Local capacity to conduct evaluations was underdeveloped, with limited knowledge of and confidence in evaluation methods.^3^

#### Standardised approaches

*'... we identified a tension between a desire to promulgate local stories of success in order to encourage the spread of innovation, and the more cautious approach embodied in the evaluation programme ... '* ^16^

Studies identified key gaps in terms of the approach to evaluation at a national level. These included the development of clear outcome measures and a shared evaluation framework,^3,6,13,15^ helping to ensure that the design of evaluation reflects the original values of the projects.^19^ These are needed from the outset of the programme; for example:

*'The impacts of the programme would have been greater if there had been more clarity regarding the expected outcomes and a better developed evaluation framework.'* ^3^

Consistent national guidance and standardised reporting frameworks would additionally help to ensure that evaluations are thorough, transparent, and in line with project objectives.^12^

### Applying what has been learnt

#### Opportunities for shared learning

‘… *make sure that what works and why is shared and that areas can learn from their mistakes*’^18^

Pilot projects are designed to test ideas and provide learning for the longer term (or for a wider roll-out). Finding ways to learn from others can contribute to effective ongoing implementation and innovation.^3,18^ National bodies can take a planned, strategic approach to facilitating shared learning,^3,6^ and peer-support networks can be designed in to transformation funding programmes to support this.^3^

Shared learning can be inhibited by differing priorities; for example, *'between a national drive to advertise successes and local need to learn from difficulties and re-configure some interventions'*.^12^ Collaborative, constructive relationships among project teams and between project teams and their evaluators need to be built up over time in order to establish transparency and trust.^12,19^

#### Receptive policy environment

'*Over time ... horizons were narrowed ... as national metrics ... became the key indicators against which the Vanguards were judged and became the basis for ongoing funding*.'^15^

This means understanding what policy and organisational factors need to be accounted for in the timeframe and financial support offered to projects, and in any national guidelines or support arrangements that are put in place alongside it; for example, *'leadership support; on-going learning; stakeholder engagement; transitional funding; and robust evaluation',* ^3^ and, *'time and headspace … for experimentation and failure'*.^18^

It may also mean continuing to value, or at least engage with, the original funding objectives. Where local objectives are supplanted by national ones part-way through implementation^15^ or where only economic factors are used to inform future commissioning decisions,^19^ it is implied that the original objectives are no longer of concern to policymakers, and valuable learning from the projects about how those objectives may be achieved could be lost or marginalised.

## Discussion

### Summary

The study set out to synthesise lessons learnt about good practice in the design, implementation, and evaluation of pilot projects testing new models of primary care, drawing on existing syntheses and reflections on project evaluations in the UK.

Factors were identified that support or inhibit success at a project and policy level. The former include working with stakeholders, including communities and front-line staff, to build a rich, contextual understanding of local needs and complexities; providing the time, space, and support necessary for the project to succeed; and setting clear objectives from the outset to support data collection, evaluation, and shared learning.

A more fundamental challenge was found at the policy level. The studies that were reviewed found that key parameters, including short-term (2–3-year) project funding, and an associated requirement to demonstrate outcomes within that timeframe, presented a major challenge. In effect, there was a mismatch between the stated aims of transformation funding (to support care redesign and better meet patient needs) and the practical effect of these parameters, which either limited projects’ ability to demonstrate success against longer-term objectives, or reduced their focus to goals that were achievable in the short term, but much less well-aligned with the original values of the project.

### Strengths and limitations

The review was limited to syntheses of evaluations of UK primary care transformation projects from 2015 onwards, and predominantly drew on grey literature. However, two of the included studies synthesise learning from >300 projects between them. Consistent themes have been identified across all included studies, which may help to inform the future development of policy in respect of new models of primary care at a national level, as well as informing local planning in response to such policies.

### Comparison with existing literature

Similar challenges were found by Lewis *et al*,^23^ who synthesised evaluations of three pilot programmes pursuing integrated care in England, including the Vanguard programme. They found a tendency for objectives to be narrowed down by decision makers over the lifetime of programmes, with particular weight given to cost-saving objectives, and a contrast between the short timeframe of the projects and the much longer timeframes that evidence suggests are required to make meaningful change.^23^ Strong leadership, shared values, time, and resources were found to be important factors supporting project success,^23^ just as the current review has found in respect of new models of primary care.

There is significant common ground between the current review's findings and those of Lewis *et al*,^23^ with both syntheses reflecting the importance of allowing sufficient time for planning, implementation, and evaluation; providing consistent support (including consistency with respect to objectives); and providing sufficient resources for a longer timeframe. These findings suggest that the challenges are not so much with finding the right ‘new model of care’, but with getting the fundamentals right, which means creating a stable environment in which projects are supported consistently, and for long enough to achieve meaningful change. Both studies also found that patient and community engagement is limited in most pilot projects. Consequently, the effect this might have on pilot project outcomes remains unknown, and should be considered a priority for any future initiatives at project and policy level.

In order for new models of care to evolve from pilots to sustained and embedded ways of working, it is important to consider the organisational context and contractual arrangements needed to support them. However, this tended not to be a core focus of the programmes in practice. In England, despite an original intention for *'the Vanguard programme* [to] *result in the development of "products" and "simple standard approaches" ... including model capitation-based contracts and service design models'*, this was not followed through in practice.^16^ In Wales, contractual arrangements were not a central focus of the Pacesetters programme; while in Scotland, negotiations for the new General Medical Services contract took place in parallel with the piloting of new models of care, meaning that, although both shared similar aims, the timing was such that the pilots could not directly inform the shape of the contract, nor vice versa.

The findings are similar to those of two reviews conducted in Canada, where financial support, leadership, and a clear articulation of the aims of policy redesign are cited as important barriers and facilitators to transformation implementation.^24,25^ The problem of short-term evaluation identified in the current analysis also echoes the findings of a recent scoping review of strategies to recruit GPs in China.^26^ The need for a standardised set of performance indicators and outcome measures for primary care transformation has also been highlighted in low- and middle-income countries elsewhere.^26,27^ Insufficiency of government funding and lack of reform coordination, which may act as barriers to project implementation across areas of varying economic development levels, has also been noted in China.^28^

### Implications for research and practice

Evaluations of primary care transformation projects found many examples of good practice, but also many barriers that prevented the translation of ambitious and inclusive aims (at the level of policy objectives and service design) into reality. The short-term nature of the funding and evaluation period was one of the most significant factors, identified across all transformation programmes, which inhibited projects’ chances of success and sustainability.^3,6,12^ This is a critical consideration for the future design of primary care transformation policies.

Data collection also emerged as a key consideration, particularly, ensuring that the right data are collected to measure population-level outcomes of interest.^19^ This may involve joining up multiple systems, or putting plans in place at the outset of a project to collect specific data. In either case, central (national) support may be required in order to achieve this at a local level, and this should be factored in by policymakers at the start of any future process. Transformation projects could also be improved by greater engagement of patients and communities.^3,6,12,18^ This depends on adequate infrastructure and appropriate skills at the local level, which need to be developed before (and continue beyond) any transformation projects, in order to be effective.^21^ Although this may benefit from national support and championing, local services could also take the lead in further developing their own approach to patient and public involvement, for the direct benefit of their communities.

Finally, the importance of evaluation was recognised in all the national transformation programmes,^3,6,12,15,18,19^ but opportunities for the different project teams to learn from each other (before, during, and after projects) were generally limited.^3,18^ Building in more opportunities for mutual support and challenge at all stages may have helped to enrich the programme, and to provide creative solutions to local challenges based on learning from elsewhere.

In conclusion, although the review has distinguished between project- and policy-level findings, policymakers may wish to take account of the former, as these may inform the factors that should be planned for at local and regional level in order to increase the likelihood of success for future policies. Likewise, people who might be involved in service redesign locally may find an understanding of the policy context helpful in recognising potential opportunities and pitfalls, and responding accordingly.

Primary care transformation requires coproduction and a rich, contextual understanding of local needs and complexities. However, a mismatch between policy objectives (care redesign to better meet patient needs) and policy parameters (short timeframes) is often a significant challenge to success. Longer timeframes, fewer and more targeted projects, and more opportunities for shared learning and support may all help to address these challenges in any future policy approaches to primary care transformation.
